# Interlaminar Shear Strength and Failure Analysis of Aluminium-Carbon Laminates with a Glass Fiber Interlayer after Moisture Absorption

**DOI:** 10.3390/ma13132999

**Published:** 2020-07-06

**Authors:** Jarosław Bieniaś, Patryk Jakubczak, Magda Droździel, Barbara Surowska

**Affiliations:** Department of Materials Engineering, Faculty of Mechanical Engineering, Lublin University of Technology, Nadbystrzycka 36, 20-618 Lublin, Poland; p.jakubczak@pollub.pl (P.J.); m.drozdziel@pollub.pl (M.D.); b.surowska@pollub.pl (B.S.)

**Keywords:** fiber metal laminates, moisture absorption, interlaminar shear strength, failure modes

## Abstract

This article presents selected aspects of an interlaminar shear strength and failure analysis of hybrid fiber metal laminates (FMLs) consisting of alternating layers of a 2024-T3 aluminium alloy and carbon fiber reinforced polymer. Particular attention is paid to the properties of the hybrid FMLs with an additional interlayer of glass composite at the metal-composite interface. The influence of hygrothermal conditioning, the interlaminar shear strength (short beam shear test), and the failure mode were investigated and discussed. It was found that fiber metal laminates can be classified as a material with significantly less adsorption than in the case of conventional composites. Introducing an additional layer of glass composite at the metal-composite interface and hygrothermal conditioning influence the decrease in the interlaminar shear strength. The major forms of damage to the laminates are delaminations in the layer of the carbon composite, at the metal-composite interface, and delaminations between the layers of glass and carbon composites.

## 1. Introduction

Among the fiber metal laminates (FMLs), the most noteworthy are the laminates where the layers are made of carbon reinforced aluminium laminates (CARALL). CARALL are characterized by impact resistance, a high fatigue strength, and a low density with very low growth rates and efficient crack bridging [1,2,3,4,5,6,7]. An important aspect of FMLs is their high resistance to corrosion. This phenomenon is caused by the composite layers acting as a barrier in the corrosion process, which is limited to the outer metal layers [1,8,9,10]. Nevertheless, the CARALL may be more prone to corrosion. In this case, corrosion resistance is especially influenced by the electrical conductivity of carbon fibers. As a result, galvanic interactions occur at the metal-composite interface, especially in wet environments [11,12,13].

The absorption of moisture may lead to a decrease in the glass transition temperature; a plasticizing matrix; swelling, cracking, and delaminations at the fiber-matrix interface, modification of the local stress state; and rupture of the adhesive bonding in the system [7,14,15,16]. It was previously shown that moisture absorption in polymer composites decreases the mechanical properties and interlaminar shear strength [17,18,19,20]. In the literature, articles on the influence of environmental conditions on the properties of fiber-metal laminates can be viewed. Botelho et al. [7] investigated the influence of hydrothermal conditioning on the mechanical properties of CARALL by conducting tensile and compression tests. The authors suggest that the changes in the mechanical properties of CARALL are negligible, similar to the case of glass reinforced aluminium (GLARE) laminates [17]. In another paper [21], the authors conducted research on hydrothermal effects by using the losipescu and interlaminar shear strength (ILSS) test for GALRE laminates. A decrease in shear strength was observed. However, it was stated that the influence of the hydrothermal effects on the shear strength results was inconsequential. The results of ILSS tests with a completely saturated GLARE specimen presented by Borgonje et al. [9] show that the presence of moisture has a significant influence on the obtained strength values. After exposure to a high-humidity environment, they observed a significant decrease in the tensile strength. In other studies, it was found that hygrothermal conditioning reduces the shear strength of CARALL laminates due to moisture absorption [5]. A decrease of the mechanical properties in CARALL after hygrothermal ageing was also observed by Pan et al. [10]. Research [22] on the effect of hydrothermal conditioning on the mechanical properties of fiber metal laminates such as GLARE and CARALL indicates a decrease in the tension resistance and ILSS of 15% and 9–11%, respectively, depending on the configuration. Ali et al. [15] investigated the seawater hydrothermal conditioning effects on the properties of titanium-carbon fiber/epoxy FMLs for marine applications. Their results showed that the percentage reduction in the mechanical properties of the Ti FML was lower in comparison to the conventional composites. The good properties of titanium fiber metal laminates after environmental conditioning in comparison to other FMLs were also confirmed by Silva et al. [23]. Poodts et al. [24] characterized a fiber metal laminate (with steel layers) for underwater applications. They observed that steel layers offer very good protection against water absorption and prevent the loss of the mechanical properties associated with it. Their results showed that saturated specimens exhibit a loss of the flexural modulus and shear strength of approximately 20%. It is very important to maintain proper bonding between the matrix and the steel layer. The authors found that water penetration at the steel-matrix interface induces delaminations, with subsequent degradation of the component. Accurate manufacturing processes ensuring a perfect bond between the steel layer and the matrix are thus strongly recommended. Singh and Angra [25] also studied the hygrothermal degradation of stainless steel fiber metal laminate conventional GF/E (glass fibre/epoxy) composites. In water, the compressive and tensile strengths of FMLs were reduced by 32.6% and 23.4%, respectively. The highest reduction in the compressive and tensile strengths of the GF/E composite was recorded as 36.8% and 29.8%, respectively, in water. Hygrothermally and unconditioned and conditioned tensile specimens fail due to delamination and fiber breakage, respectively, while compression specimens fail because of delamination between GF/E layers. Park et al. [26] investigated the effects of void contents on the long-term hydrothermal behaviors of GLARE laminates. They observed that the voids contribute to the water absorption properties and that the decrease in ILSS depends on the characteristic of water adsorption and voids. Large ILSS degradations of between 17% and 32% occurred for GLARE in comparison to the non-aged counterparts. Chandrasekar et al. [27] presented an experimental review on the mechanical properties and hydrothermal behavior of fiber metal laminates. Botelho et al. [28] also researched the hydrothermal effects on viscoelastic properties, such as the storage modulus (*E*′) and loss modulus (*E*″), for glass fiber/epoxy/aluminum laminate (GLARE). For GLARE laminates, the *E*′ modulus remains unchanged (49 GPa) during the cycle of hydrothermal conditioning. The research conducted by Zong et al. [29] showed a significant decrease in both the fatigue life and the tensile strength of Glare 4A laminates after being hygrothermally aged. Hu et al. [30] studied the long-term moisture absorption behavior of carbon-fiber reinforced polyimide (CF/P) and polyimide–titanium-based fiber metal laminates. The fiber metal laminates exhibited a slower moisture absorption rate in comparison to the pure composite. This was a result of the barrier function of the metal layers at both surfaces of the fiber metal laminates. The interlaminar and flexural strength of CF/P and fiber metal laminates decreased after hygrothermal ageing exposure. The mechanical properties of CF/P composites were reduced more than those of the fiber metal laminates. In the fiber metal laminates, debonding was only observed in the edge area. Molerio et al. [31] developed a new layer-wise mixed model for a coupled hygro-thermo-mechanical static analysis of fiber metal laminates, under a series of hygro-thermo-mechanical loadings. In another work, Molerio et al. [32] presented three-dimensional exact hygro-thermo-elastic solutions for multilayered plates: composite laminates, fiber metal laminates, and sandwich plates. These effects are demonstrated by the through-thickness distributions of displacements, stresses, temperatures, and weight percent moisture contents for the selected multilayered plates under different hygro-thermo-mechanical loadings, which may serve as 3D benchmark exact solutions. Works focused on improving the interlaminar strength can also be noted. For example, Garcia and co-authors [33] studied the effect of nylon nanofibers on the delamination resistance and dynamic behavior of GFRP composites. Due to the incorporation of nylon nanofibers in the nano-modified composites, a significant (27%) increase in the inter-laminar shear strength was obtained. This can be related to the presence of significant bridging phenomena due to the interaction between the nylon nanofibers and the epoxy resin. The authors concluded that improvement of the delamination resistance of GFRP laminates can be obtained by using nylon nano-fibers.

In general, despite a significant resistance to environmental factors, moisture absorption may significantly influence the metal-composite interface in the process of degradation, as well as corrosion. It is also known that in fiber metal laminates, the interface between the aluminium and fiber reinforced polymers layer plays an important role in the stress transfer mechanisms of FML laminates [21]. Moreover, a significant damage process at the metal-composite interface, along with the occurrence of cracks and delaminations, is observed during the influence of moisture on fiber metal laminates. One of the methods used to increase the corrosion resistance in fiber metal laminates with carbon fibers is protecting the metal-composite interface as a result of isolating the composite layers from the aluminium layers. This is achieved by introducing thin thermoplastic layers (PEI, polyetheroimide) or GFRP between the aforementioned layers as electrical insulators [1]. Moreover, research on the application of additional hybrid sol-gel layers to the metal in order to increase its corrosion resistance and introducing an additional layer of epoxy resin at the metal-composite interface has been conducted [7]. Applying insulating interlayers, such as PEI or glass fiber layers, may cause a change in the mechanical properties of the FMLs.

To the best of the authors’ knowledge, the behavior at the interface of a hybrid system Al-glass fiber-carbon fiber under the influence of environmental conditions has not been reported in the open literature. Therefore, there is a need to conduct research in order to improve our understanding of the environmental influence on the behavior of fiber metal laminates, which should be included in the process of designing composite structures. This paper presents results on the interlaminar shear strength of hybrid fiber metal laminates before and after environmental conditioning. The objective of this study was to conduct an analysis of the influence of an additional layer of glass fiber composite, applied between the aluminium and carbon fiber composite. Moreover, the characteristics of the types and mechanisms of damage as a result of the simultaneous influence of the moisture and an elevated temperature, including an analysis of the metal-composite interface, are presented.

## 2. Experimental Analysis

### 2.1. Material

The subjects of the research were fiber metal laminates based on aluminum alloys and fiber reinforced polymers. The FMLs were manufactured by using 2024-T3 aluminium alloy (thickness of 0.3 mm) and unidirectional AS7J high-strength carbon and R-glass fiber reinforced epoxy resin prepregs (Hexcel, Stamford, CT 06901, USA). The nominal fiber content was about 60 vol.%. In order to ensure better adhesion of the components, the surfaces of aluminum sheets were anodized in chromic acid (CAA) and coated with EC 3924B corrosion inhibiting structural adhesive primer (3M, St. Paul, MN, USA). The anodizing process was carried out in accordance with the procedures used in the aviation industry. The process consisted of a number of stages, including rinsing, drying, alkaline degreasing, and sulffochromic etching. Anodizing was performed in CrO_3_ chromic acid anhydride at a temperature of 40 °C, a voltage of 20 V, and a time of about 45 min. After anodizing, another surface preparation step was carried out within 8 h. It consisted of applying a thin layer of epoxy-based, synthetic resin primer (EC 3924B, 3M, St. Paul, MN, USA) containing a corrosion inhibitor. Such a primer promotes long-term durability for bonded joints by creating an interface between the adhesive and the metal. The primer curing cycle was 30 min at 24 °C; then, the process was repeated at 121 ± 5 °C for 60 min.

**Figure 1:**
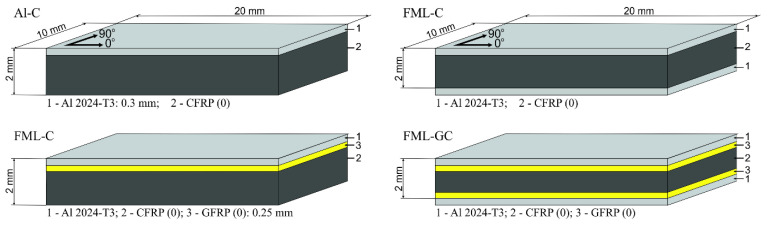
The configurations of the tested laminates.

Four different types of laminates were manufactured: aluminium/CFRP (Al-C), aluminium/CFRP with a GFRP interlayer (Al-GC), 2/1 FML with CFRP (FML-C), and 2/1 FML with a CFRP and GFRP interlayer (FML-GC). The configurations of the tested laminates and dimensions of the samples for ILSS and hydrothermal conditioning are shown in Figure 1.

The unidirectional laminate configuration used is typical of the ILSS test. In 2/1 FML laminates, moisture only occurred through the free side edges. However, the one-sided configuration allowed the determination of the behavior at the metal-composite interface with a significantly larger moisture absorption surface by the composite. This can be a representation of metal-composite adhesive joints. The laminates were produced by the autoclave method (Scholz Maschinenbau, Coesfeld, Germany). The cure cycle was carried out at a heating rate of 2 °C/min up to 135 °C and held at this temperature for 2 h. The pressure and the vacuum used were 0.4 and 0.08 MPa, respectively.

### 2.2. Hygrothermal Conditioning

**Figure 2:**
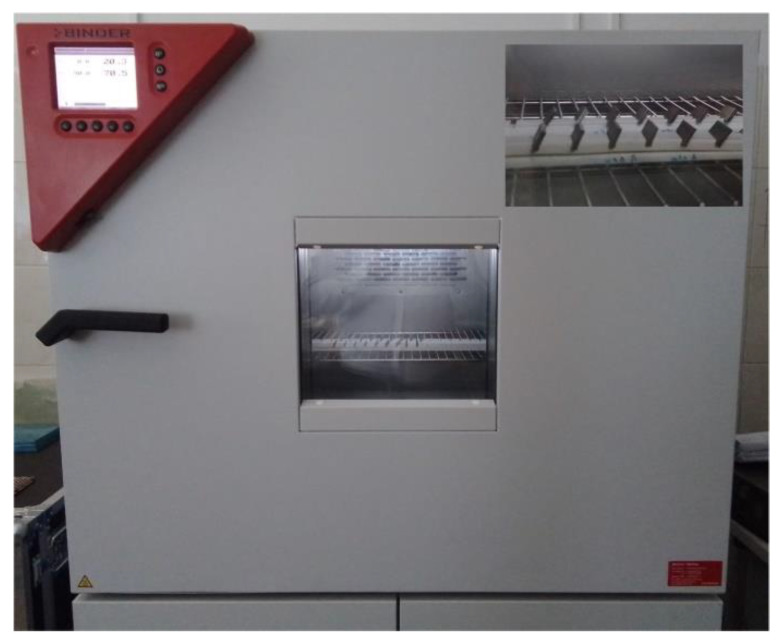
The environmental chamber used for hygrothermal conditioning.

In order to assess the influence of the hygrothermal conditioning on the shear strength, the fiber metal laminate specimens were exposed to a combination of temperature and humidity in an environmental conditioning chamber according to ASTM D 5229 [34]. During the initial stage, the specimens were subjected to drying at 70 ± 1 °C for a period of 10 days in a laboratory drying oven (SLW 53ECO, Pol-Eko, Wodzisław Śląski, Poland), in order to remove moisture and determine the initial state of the hygrothermal conditioning process. The specimens were then conditioned at the temperature of 60 °C and a 99% relative humidity to the point of reaching moisture equilibrium. Hygrothermal conditioning tests were performed using the Binder automated environmental chamber (MKF 115, Binder, Tuttlingen, Germany, see Figure 2).

Changes in the mass of the control samples were registered periodically at 24 h intervals (consecutively every few days) with a laboratory scale (Radwag WAS 220/X) to an accuracy of 0.0001 g. The duration of the test was 100 days. Then, samples after this exposure time were subjected to strength tests.

### 2.3. ILSS Test

**Figure 3:**
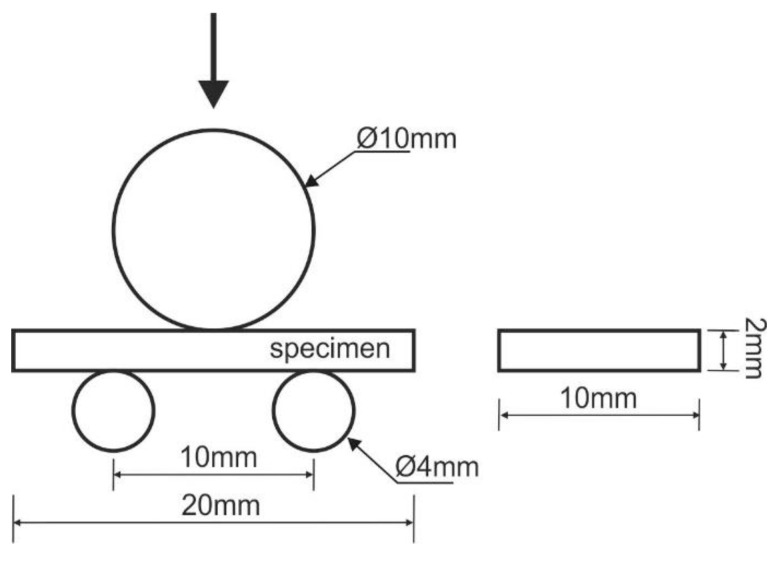
Schematic configurations of the short beam shear test specimen.

The interlaminar shear strength (ILSS) was determined by using the short beam shear test according to EN ISO14130:1997 [35]. Ten specimens were tested for each type of stacking sequence, in order to assess the effect of environmental conditions on the ILSS. The detailed test and major dimensions are presented in Figure 3.

The tests were performed in a Schimadzu mechanical testing machine using a test speed of 1.3 mm/min. The interlaminar shear strength (ILSS) was determined by the following Equation [35]:

(1) $$\tau =\frac{3F}{4bh}\;\left[\mathrm{MPa}\right],$$

where *F* is the maximum load, and *b* and *h* are the width and thickness of the specimen, respectively.

### 2.4. Fractography

The tested specimens after the short beam shear test were examined under the scanning electron microscope (SEM) for the characterization of failure modes due to the combined effect of moisture absorption and an elevated temperature. An FEI NovaNano SEM 450 field emission scanning electron microscope was used for SEM analysis.

## 3. Results and Discussion

### 3.1. Moisture Absorption

**Figure 4:**
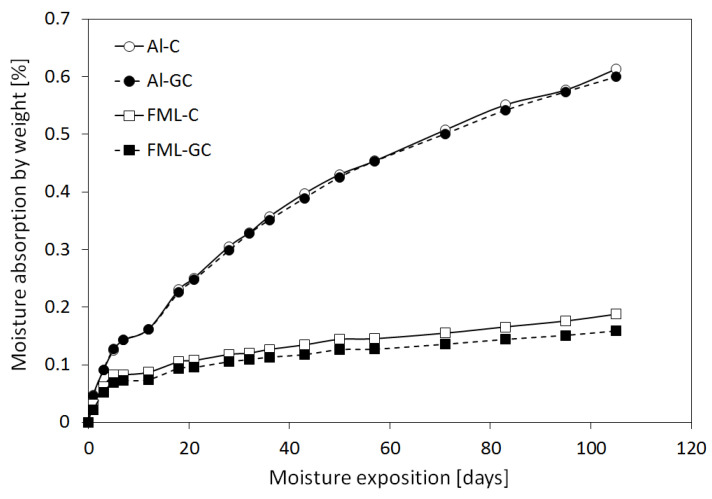
Moisture absorption of the materials studied.

Figure 4 presents the moisture absorption for one-sided laminates (Al-C and Al-GC) and FML laminates (FML-C and FML-GC) without and with a glass composite layer subjected to exposure at 60 °C and 99% RH.

It can be observed that moisture absorption in FML laminates is insignificant in relation to one-sided laminates (Figure 4). A constant increase of moisture adsorption in the examined laminates can be observed during the entire exposure time. Moreover, it can be noticed that a typical equilibrium point does not occur in this case. This indicates that in the absorption process, anomalies and non-Fickan behavior occur. For both investigated Al-C and Al-GC laminates, the maximum moisture absorption during hygrothermal conditioning reached 0.6 mass %. In the case of FML-C and FML-GC, it can be observed that after c.a. 100 days, laminates absorb moisture at the level of 0.18% (see Figure 4). It was also noted that FML-GC absorbs slightly less moisture (0.15%) compared to the laminates with a carbon fiber composite (FML-C). A significantly lower moisture absorption index in fiber metal laminates in comparison to one-sided laminates is caused by the outer metal layers [5,9,10]. Those aluminium layers protect FMLs from moisture absorption by decreasing the surface of the laminate open to moisture. For this reason, moisture diffusion processes in fiber metal laminates only occur through free edges of the specimens (side edges). Nevertheless, due to absorption through free edges, the moisture distribution in the laminate may not be uniform. The literature data [36] indicates that the increase of the FML mass at the saturation point only constitutes 10% of the increase for traditional composite materials (GFRP/CFRP). As a result, the effect of hygrothermal conditioning in FMLs will be much less significant than in the case of conventional composites.

It is known that a disadvantage of polymers, such as epoxies, is the fact that they are prone to absorb moisture during exposure to humid environments because the epoxy matrix is highly polar [26]. Moisture absorption from the atmosphere generally occurs through the surface, followed by the diffusion process through most of the material [5,7]. Water molecules can create hydrogen bonds with the molecular structure of the matrix during absorption. The O-H dipole in the water molecule attracts hydroxyl (O-H), amine (N-O), sulphone (O-S), and phenol (O-C) groups. In the long term, these chemical reactions deteriorate the resin by hydrolysis [37]. On the other hand, as a result of capillary action, moisture can be transported to the bulk of the composite specimens by microcracks at the fiber-matrix interfaces [5,7,38]. The fiber-matrix interfaces are responsive to the chemical effect of water because the glass contains alkaline oxides that can be hydrolyzed. The mechanical properties of the glass are reduced by the water, which hydrates these oxides [37].

As far as the fiber metal laminates are concerned, the metal surface with an oxide layer also ought to be considered. In the case of metal, where the oxide layer is formed on the surface, hydration of this layer may occur due to the moist environment. In turn, low-adhesive aluminum hydroxide is created [15,17,21,26]. Moreover, it causes a significant increase in volume, leading to higher stresses around the part-through crack tip and subsequently to higher crack growth rates [9]. In the case of the investigated FML laminates, the aluminium surface was prepared by anodizing and applying primer. Such an action improves the adhesion and corrosion resistance between the composite and the aluminium [10]. For this reason, due to stable, adherent, and continuous oxide film [10], one can expect the anodized material layers to absorb less moisture. Anodizing pretreatments such as chromic or phosphoric anodizing demonstrated a stable oxide layer, most probably resulting from the presence of ions (e.g., PO_4_ for the phosphoric anodizing process) at the surface which prevent oxide hydration. Another reason for an improved durability of the anodized oxide layers may be the absence of certain elements (e.g., magnesium) in the oxide layer [39]. Additionally, the primers mostly contain chromate inhibitors, which limit the moisture diffusion rate through the primer [39].

In the case of conventional composites, and perhaps especially FMLs, where absorption mostly occurs through side edges, the influence of reinforcing fibers should be considered. Moisture absorption by reinforcing fibers is inconsequential, so they work as moisture barriers. However, applying those fibers as a reinforcement in composites causes important changes in the diffusion pathways of water molecules in an anisotropic way, hindering simple diffusion [18]. The varied angular positioning of the fibers may influence changes to the character of the composite diffusion by increasing or decreasing the diffusion length of water molecules [18]. Combined with capillary forces acting in the direction of the fibers, this causes a much higher moisture absorption rate in the direction of the fibers than perpendicular to this direction [9].

### 3.2. Interlaminar Shear Strength

**Figure 5:**
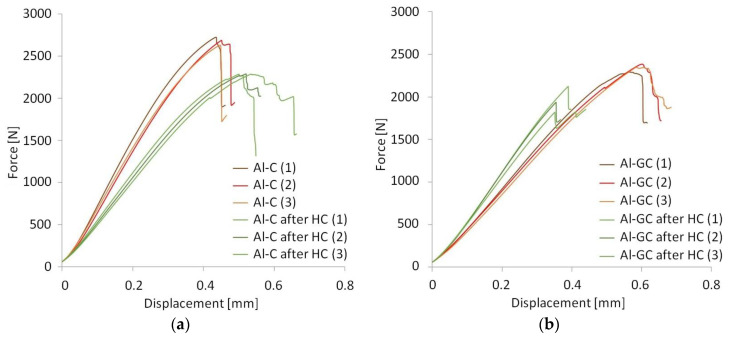

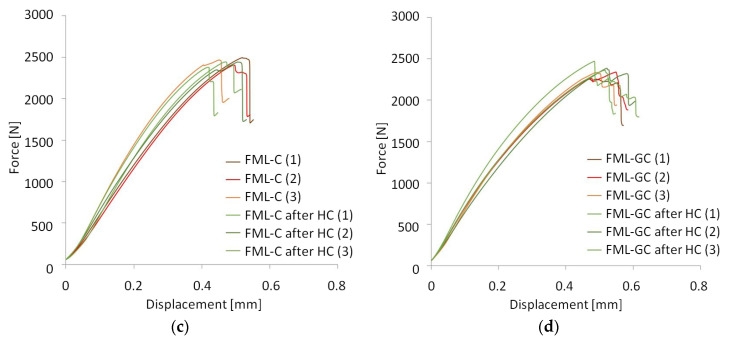
Force–displacement curves of fiber metal laminates (FMLs) after interlaminar shear strength (ILSS) tests: (**a**) Al-C, (**b**) Al-GC, (**c**) FML-C, and (**d**) FML-GC.

Figure 5 shows the typical force–displacement curves of the examined laminates. The force–displacement curves look similar for the investigated laminate groups. Upon reaching the maximum load, each curve shows sharp load drops, demonstrating that complete interlaminar failure has taken place.

Table 1 shows the interlaminar shear strength results for one-sided and FML laminates with and without a layer of glass composite. The obtained results show that ILSS depends on both the laminate configuration and hygrothermal conditioning.

The data presented in Table 1 shows that the interlaminar shear strength for Al-C without an additional layer of glass composite is higher than that for the Al-GC laminate containing a layer of glass laminate (93 vs. 86.4 MPa, respectively). Similarly, for FML-C and FML-GC, it can be observed that the laminates without the layer of glass composite reach slightly higher values of ILSS (84.6 vs. 81.5 MPa, respectively). Upon comparing the interlaminar shear strength in both laminates (one-sided and two-sided laminates) with and without the layer of glass composite before hygrothermal conditioning, higher ILSS values can be noted for one-sided laminates (see Table 1). It can be assumed that introducing additional layers of both metal and glass composites decreases the interlaminar strength. This phenomenon may be caused by an increase of aluminium, carbon, and glass composite interfaces. Upon analysing the influence of hygrothermal conditioning, it can be observed (see Table 1) that it does not cause the interlaminar shear strength to decrease for both FML-C and FML-GC laminates. In the case of one-sided laminates (Al-C and Al-GC), a significant decrease in the interlaminar shear strength after hygrothermal conditioning can be observed. For the Al-C laminate without an additional layer of glass composite, the decrease in the interlaminar shear strength after hygrothermal conditioning oscillates around 11%. For the Al-GC laminate with a layer of glass composite, a decrease of 23% was noted. Upon comparing the influence of the configuration of one-sided laminates after hygrothermal conditioning, it was noted that in the case of laminates with an additional layer of glass composite, the interlaminar shear strength decreases by 19% in comparison to laminates without the glass composite layer. According to [5,17,23,40], moisture absorption by polymer composites may cause the resistance and stiffness of laminates to decrease, which is caused by a significant plasticizing effect on the matrix with the weakening of the fiber-matrix distribution. Moreover, the water molecules enter the molecular structure, which generates residual stresses and causes the matrix to swell [37]. Therefore, as observed in this research, plasticization of the matrix and laminate distribution surfaces leads to a decrease in the interlaminar shear strength values due to hygrothermal conditioning.

### 3.3. Failure Analysis

**Figure 6:**
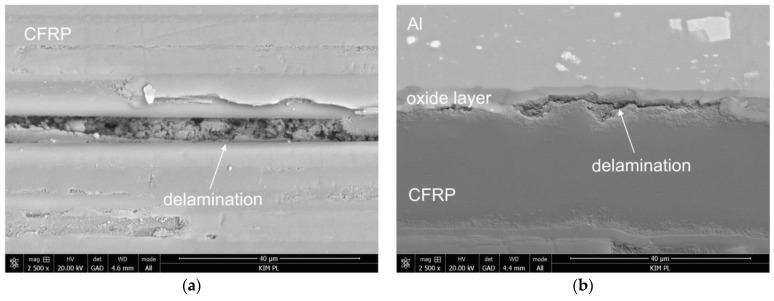
Types of damage in one-sided laminates (Al-C) before (**a**) and after (**b**) hygrothermal conditioning.

**Figure 7:**
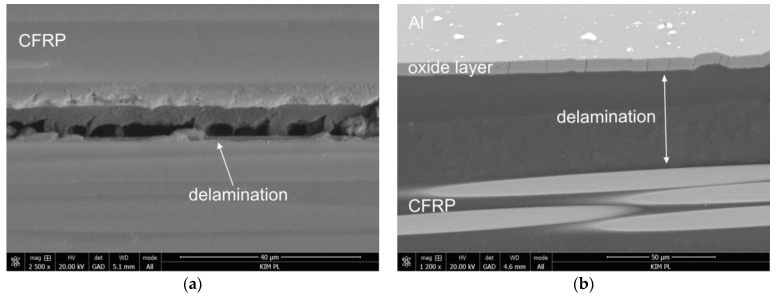
Types of damage in one-sided laminates with an additional layer of glass composite (Al-GC) before (**a**) and after (**b**) hygrothermal conditioning.

**Figure 8:**
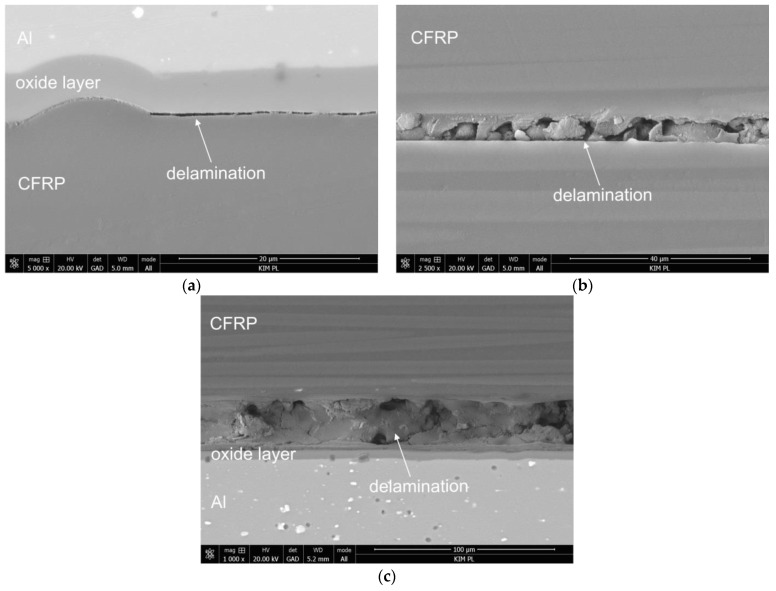
Types of damage in FML laminates without an additional glass composite layer (FML-C) before (**a**), (**b**) and after (**c**) hygrothermal conditioning.

**Figure 9:**
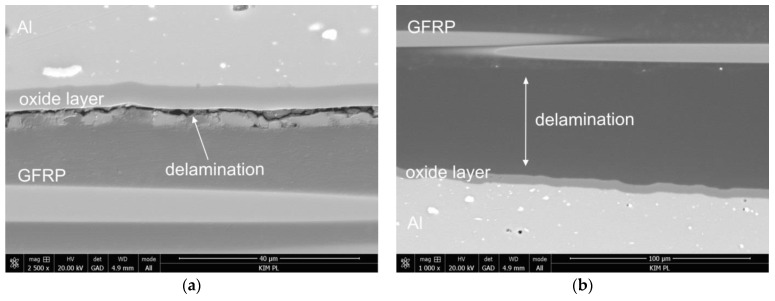

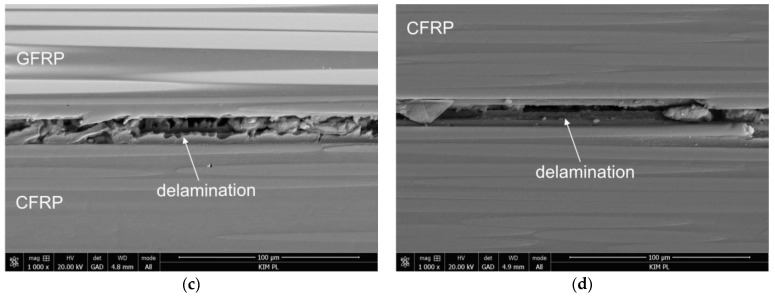
Types of damage in FML laminates with an additional layer of glass composite (FML-GC) before (**a**–**c**) and after hygrothermal conditioning (**d**).

In order to identify the type of damage in the investigated laminates before and after hygrothermal conditioning, a microstructure analysis was conducted. Figure 6, Figure 7, Figure 8 and Figure 9 show characteristic forms of damage to the laminate microstructure. In the case of one-sided laminates without an additional layer of glass composite (Al-C), it was observed that the characteristic form of damage occurring before hydrothermal conditioning is only delamination in the carbon composite layer (Figure 6a). Alternatively, after hygrothermal conditioning, delaminations in both the layer of carbon composite and at the metal-composite interface are observed (Figure 6a,b).

The analyses of the damage of Al-GC laminates with an additional layer of glass composite indicate that, similar to the above presented case before hygrothermal conditioning, the delaminations only occur in the carbon composite layer in the area of the indenter (Figure 7a). After hygrothermal conditioning, significant delaminations can be observed between composite layers and the metal and the cracks of the oxide layer (Figure 7a,b). Moreover, delaminations in the central area of the carbon composite layer were noted.

In the case of two-sided laminates without an additional layer of glass composite (FML-C) before and after hygrothermal conditioning, a few small delaminations at the interface of the oxide layer and composite layer, as well as delaminations in the carbon composite layer, were observed (Figure 8a,b). It can also be observed that significant delaminations occurred at the metal-composite interface in the area of the indenter (Figure 8c).

Upon analysing the damage to the FML-GC laminates with an additional layer of glass composite (FML-GC) before hygrothermal conditioning, delaminations at the metal-composite interface were observed. Moreover, these delaminations were observed in both the upper and lower part of the laminate in the area of the indenter (Figure 9a,b). Additionally, delaminations on the surface of the distribution between the glass and carbon composites, as well as delaminations in the carbon composite (Figure 9c,d), were noted. After hygrothermal conditioning, however, only delaminations in the carbon composite in the area of the indenter were observed (Figure 9c).

The behavior of laminates subjected to shear is a property dominated by the matrix. The interlaminar shear strength is controlled by an interface between the fiber and matrix in the case of a composite, while in the case of FMLs, it is controlled by the interface between the metal and the composite layer [5,36]. The fractographic analyses performed may indicate that the metal-composite and fiber-matrix interface is susceptible to the chemical action of moisture. In the case of interlaminar shear, the moisture effects on composites affect the mechanical behavior of the resin and the distribution of interlaminar stresses [5]. Stresses resulting from hygrothermal environments generally reach the maximum value at the fiber-matrix interfaces and boundaries between adjacent composite layers of different fiber orientations [29]. The interface between the reinforcing fibers and the epoxy matrix, as well as between the oxide layer and epoxy matrix, plays a significant role in the effective transfer of loads in a composite. The decrease in the interfacial connection strength due to the weakening of the interface would hinder the effective transfer of loads. Unfortunately, the fiber-matrix interfaces are sensitive to the chemical effects of water. The glass contains alkaline oxides which can be hydrolyzed, possibly leading to permanent changes in the material and hence reductions in the mechanical properties of the fibers. The epoxy resin only adheres to untreated glass fibers by hydrogen bonding, while treated glass fibers form chemical and hydrogen bonds with epoxy. At the interface, voids are created because water easily debonds hydrogen bonds [9].

Residual stresses in laminates are caused by swelling and plasticizing of the epoxy matrix as a result of moisture absorption. This may cause interlaminar shear deformation, which causes micro-crack formation in the interlayers and the initiation of micro-cracks on the interface of the fiber-matrix and at the metal-composite interface, and as a result, ILSS reduction [15,18]. In good fiber-matrix interfacial adhesion systems, the swelling effect is not significant.

In the case of the metal-composite interface, the moisture preferentially diffuses towards the oxide layer because both the oxide and the water are more polar. In this respect, the resin boundary layer near the interface may be important in the diffusion behavior. It is certain that the physical and mechanical properties of this resin boundary layer will differ from those of the bulk materials. However, on this issue, there is an interesting discrepancy in the literature. It is shown that this resin boundary layer is more susceptible to hydrolysis than the bulk resin because of its lower cross-link density, whereas other authors consider the boundary layer to be a diffusion barrier because of its tighter cross-linking [39]. The authors of [41] also observed that fiber-matrix debonding mainly occurred near layer boundaries, where evidence for excessive shear deformation was found. They concluded that this is a result of a resin-rich phase near the layer boundaries. This phenomenon allows for higher shear deformation when the stress state is higher than the resin yield stress.

In sum, the damage analysis carried out in this work indicates the complexity of the damage process in fiber metal laminates.

## 4. Summary

This paper has presented aspects of the interlaminar shear strength of hybrid fiber metal laminates with a carbon composite before and after hygrothermal conditioning. In this study, the aspects of the metal-composite interface of hybrid laminates with an additional layer of glass composite were discussed. The influence of environmental factors, the interlaminar shear strength, and the failure mode were researched and analysed. On the basis of the conducted research, as well as the results of the influence of moisture and temperature, it was stated that the fiber metal laminates can be counted among the materials with lower moisture adsorption compared to conventional composite materials. This phenomenon is caused by the characteristic structure of fiber metal laminates and a limited area of intensive moisture absorption on the side surfaces of the laminate. The insertion of an additional layer of glass composite decreases the interlaminar shear strength in both one- and two-sided laminates. This can be caused by the material configuration and varied properties of the selected materials: metal, carbon fiber composite, and glass fiber composite. Hygrothermal conditioning has a detrimental effect on one-sided laminates, as opposed to the two-side type of laminates, resulting in a decrease of the interlaminar shear strength. The fractographic analysis indicates that the main forms of damage in both one- and two-sided laminates are delaminations. They were observed in the carbon composite layer and at the metal-composite interface. In the case of laminates after hygrothermal conditioning, they were also observed between the layers of glass and carbon layers. It was confirmed that each type of interface in laminates is sensitive to interfacial debonding by the shear load.

Further research and the development of potential alternative strategies to protect the metal-composite interface appear necessary due to the negative impact of moisture phenomena. It is necessary to prepare a surface with an appropriate metal-composite interface. High adhesive properties are required, especially in conditions of long-term exposure to moisture. This can be achieved by creating a stable oxide layer and using additional intermediate layers, e.g., a primer with a corrosion inhibitor. Sol-gel layers are currently the preferred alternative for metal surface preparation, ensuring adequate adhesion on the metal-composite interface. These layers also have a pro-ecological aspect. Another solution may be the use of composite matrix systems with low moisture absorption, especially at elevated temperatures. An important alternative may also be the use of nanomodified composites containing nanofibers (e.g., nylon), graphene, or carbon nanotubes to increase the interlaminar shear strength.

## Figures and Tables

**Table 1:** Interlaminar shear strength values for tested laminates.

**Laminate Type**	**Before Hygrothermal Conditioning**			
	**Al-C**	**Al-GC**	**FML-C**	**FML-GC**
F_max_ [N]	2728 (±125 *) CV ** = 4.59	2374 (±69) CV = 2.91	2366 (±84) CV = 3.55	2326 (±40) CV = 1.72
ILSS [MPa]	93.0 (±2.3)	86.4 (±2.6)	84.6 (±2.4)	81.5 (±1.7)
**Laminate Type**	**After Hygrothermal Conditioning**			
	**Al-C**	**Al-GC**	**FML-C**	**FML-GC**
F_max_ [N]	2299 (±90) CV = 3.91	1991 (±130) CV = 6.54	2400 (±82) CV = 3.44	2351 (±75) CV = 3.18
ILSS [MPa]	82.3 (±2.7)	66.8 (±4.1)	85.9 (±2.0)	81.8 (±1.4)

* Standard deviation and ** sample coefficient of variation, in percent.

## Data Availability

The raw/processed data required to reproduce these findings cannot be shared at this time as the data also forms part of an ongoing study.
